# Effect Of Different Doses Of Noradrenaline Against Ischemia-induced Ventricular Arrhythmias In Rat Heart In Vivo

**Published:** 2009-01-01

**Authors:** Alireza Imani, Mahdieh Faghihi, Mansoor Keshavarz, Seyed Morteza Karimian, Somayeh Sadeghi Niaraki

**Affiliations:** Department of Physiology, School of Medicine, Tehran University of Medical Sciences, Tehran, I.R. Iran

**Keywords:** arrhythmias, noradrenaline, ischemia, preconditioning

## Abstract

**Background:**

The present study was designed to evaluate the preconditioning effect of different doses of noradrenaline on ischemia-induced ventricular arrhythmias in open chest anesthetized rats.

**Methods:**

The anaesthetized rats were subjected to 25 min of regional ischemia by left descending coronary artery (LAD) occlusion. In sham group, surgical procedures were done but ischemia was not applied. In control rats, saline was injected prior to ischemia. In noradrenaline groups, rats pretreated with three different doses of noradrenaline (respectively, 0.5, 1 and 2 μg/kg, IV).

**Results:**

In control rats, induction of ischemia shortened the QTc (corrected QT) interval (ms) and led to occurrence of ventricular arrhythmias. Administration of low-dose of noradrenaline prevented shortening of the QTc interval during ischemia but could not significantly attenuate severity and incidences of arrhythmias. Injection of mid-dose of noradrenaline stabilized the QTc during ischemia and reduced severity of arrhythmias. Pretreatment with high-dose of noradrenaline significantly prolonged the QTc interval and declined severity and incidence of arrhythmias.

**Conclusions:**

Noradrenaline dose-dependently attenuated ischemia-induced ventricular arrhythmias.

## Introduction

The heart can protect itself against prolonged ischemic injury by prior exposure to one or more short-lasting periods of ischemia. This phenomenon, known as ischemic preconditioning (IPC) [1], presents one of the most effective manners for reducing the myocardial injury.

Although preconditioning limits the extent of infarct size as a gold standard used in animal studies [2], its effect on arrhythmias is less clear with opposing results ranging from inhibition of arrhythmias [3] to its worsening [4].

One of the ways to achieve IPC-like cardioprotection is the stimulation of a1-adrenoceptors by catecholamines. In this regard, there are controversial reports from the effect of α1-adrenoceptor stimulation on ischemia-induced ventricular arrhythmias in the isolated rat heart. It has been shown that stimulation of this receptor worsens arrhythmias [5], whereas another study has indicated that transient stimulation of these receptors inhibits ischemia-induced ventricular arrhythmias [6]. In addition, it has been shown that sympathetic activation triggers life-threatening arrhythmias in rat heart [7].

Many factors, which are absent in the isolated heart but present in vivo (e.g. hormonal and/or autonomic neural effect, circulatory system and loading of right ventricle) may also be important in the effect of catecholamines on ischemia- induced ventricular arrhythmias.

However, ethical considerations confine the experimental work on the human heart to assess the cardioprotection effect of preconditioning against ischemia injuries, the possibility that a myocardial protection might be inducible in the human heart has been led to experimental research on animals and it can only be attained when carefully planned clinical studies have been carried out in suitable patients with risk of coronary artery occlusion [8]. In the clinical setting, there is some evidence to suggest that preconditioning may occur in patients with coronary artery disease [8-11]. Patients suffering angina before a myocardial infarction (MI) have a better in-hospital prognosis and a reduced incidence of life-threatening ventricular arrhythmias [9]. Previously, the latent protection conferred by ischemic preconditioning has been shown in the clinical setting that led to reduce the complications associated with the procedures such as precutaneous transluminal coronary angioplasty (PTCA) [10] and coronary artery bypass surgery (CABG) [11].

Therefore, the present study was designed to assess the preconditioning effect of different doses of exogenous administration of noradrenaline (the predominant transmitter of cardiac sympathetic nerve) on ischemia-induced ventricular arrhythmias in open chest anesthetized rats.

## Methods

### Surgical Preparation

Male wistar rats weightening 250-350 g (12-16 weeks old) were anesthetized by intraperitoneal administration of pentobarbital sodium (50 mg/kg body weight). The animal care was conducted in accordance with the institutional guidelines of Medical Sciences University of Tehran (I.R.) and the National Institutes of Health (NIH) guidelines for the care and use of laboratory animals. Surgical preparation to induce ischemia was performed by the following method [12,13]:

The rats were tracheotomized in middle of the neck, intubated and ventilated with room air by a Parvalux rodent ventilator (15 ml/kg stroke volume and 60-70 breaths/min). Body temperature was measured with a rectal thermometer and maintained at 37 ±1ºC by the use of a lamp. The right carotid artery was cannulated and connected to a pressure transducer to measure mean arterial blood pressure (MBP). The tail vein was cannulated to allow administration of saline or drugs. A standard limb lead-II electrocardiogram (ECG) was monitored with subcutaneous stainless steel electrodes. ECG and MBP were recorded and heart rate was calculated using the Power Lab monitoring system (ML750 PowerLab/4sp). Rats were given heparin (200 IU/ kg, IV), and then the chest was opened by a left thoracotomy in the fifth intercostal space to expose the heart. After incision of the pericardium, a 6-0 silk suture was placed around the left anterior descending coronary artery (LAD), close to its origin. Both ends of a silk thread were passed through a polyethylene tube. Applying tension to the suture caused regional ischemia following coronary artery occlusion, and reperfusion was achieved by releasing the tension on the ligature. Ischemia was confirmed by ST elevation and increase in R-wave amplitude in ECG, or cardiac cyanosis. At the end of surgical procedure, any rat with a constant fall in MBP to less than 80 mm Hg or ventricular fibrillation lasting for more than 5 min was discarded from the study. The end points used in this study were the severity and incidences of ventricular arrhythmias.

### Assessment of ventricular arrhythmias

**Figure 1:**
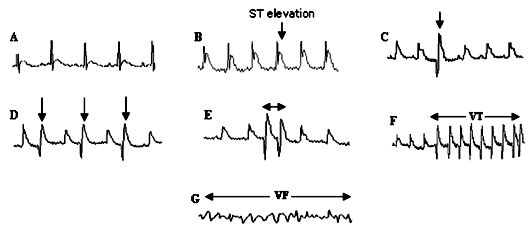
Electrocardiogram recording. A: during baseline. B: during coronary artery occlusion. C: ventricular ectopic beat (VEB). D: bigeminy. E:couplet. F: ventricular tachycardia (VT). G: ventricular fibrillation (VF)

Ischemia-induced ventricular arrhythmias were determined in accordance with the Lambeth Conventions [14]. Ventricular ectopic beats (VEBs) were defined as identifiable premature QRS complexes. Ventricular tachycardia (VT) was defined as the occurrence of four or more consecutive VEBs at a rate faster than the resting sinus rate. Ventricular fibrillation (VF) was defined as unidentifiable and low voltage QRS complexes. In the case of other multipart forms of VEBs such as bigeminy, couplet (two consecutive VEBs) and triplet (three consecutive VEBs), they were counted at separate episodes (Figure 1).

Ventricular fibrillation may be sustained or may revert spontaneously to a normal sinus rhythm. VF lasting for more than 5 min was considered as irreversible.

A scoring system was used to calculate the severity of arrhythmias  [15] where hearts with 0-50 VEBs were given score 0; 50-500 VEBs a score of 1; more than 500 VEBs, or one episode of spontaneously reversible VT or VF a score of 2; 2-30 episodes of spontaneously reversible VT and/or VF a score of 3; more than 30 episodes of spontaneously reversible VT and/or VF a score of 4 and irreversible VF was given a score of 5.

### Evaluation of QT interval

The ECG tracings were analyzed visually and the following ECG parameters were examined:

1. RR interval (the interval between the apex of two consecutive adjacent R waves),
2. QT interval (the interval between beginning of Q wave and T wave apex), and
3. Corrected QT interval (QTc), defined as the QT interval corrected for the heart rate by means of Bazett's equation: QTc = QT (ms)/[RR (s)]^1/2^ [16].

### Materials

Pentobarbital sodium and noradrenaline were obtained from Sigma Chemical Co. These chemical agents were dissolved in saline immediately before use.

### Experimental protocols

All animals underwent a 25 min of coronary artery occlusion. Drugs were injected intravenously. After a stabilization period following the surgical preparation, rats were divided into five experimental groups.

Group-1: Sham-operated (n=9). Rats underwent surgical procedures without coronary artery ligation.

Group- 2: Control (n=9). Saline was given 10 min before coronary artery occlusion.

Group-3: NA-0.5 (n=6). Noradrenaline (0.5 μg/kg, i.v.) was administrated 10 min before coronary artery occlusion

Group-4: NA-1 (n=6). Noradrenaline (1 μg/kg, i.v.) was administrated 10 min prior to 25-min ligation.

Group-5: NA-2 (n=9). Noradrenaline (2 μg/kg, i.v.) was administrated 10 min before coronary artery occlusion.

It is mentionable that different doses of NA were chosen by preliminary study.

### Statistical analysis

Data are expressed as means±SD or the percentage of incidence. The parametric data were tested using the method Kolmogorov-Smirnov (KS). Statistical comparison of means between groups was performed by one-way ANOVA and a subsequent tukey test.

Within each group, differences between means in hemodynamic parameters were compared by one-way repeated measures ANOVA and a subsequent Bonferroni test. Differences between means in the QTc intervals were compared by paired t test. The incidences of VT or VF were compared using the Fisher exact test and the arrhythmia scores were analyzed with Kruskal-Wallis test. Significant differences were determined as P<0.05.

## Results

### Hemodynamic

MBP and HR were continuously recorded during the experiments and calculated during the 15-min baseline, preocclusion, and 25-min ischemia periods. No significant differences were found in HR and MBP between groups during baseline.

Injection of different doses of noradrenaline caused a rapid and transient increase in MBP and HR, but they had returned to the baseline levels prior to LAD ligation. (Administration of low-dose of noradrenaline resulted in a non-significant increase MBP, and injection of either the mid-dose or the high-dose of noradrenaline caused non- significant fall in MBP in preocclusion period when compared with baseline). The hemodynamic changes are outlined in Table 1.

### Ventricular arrhythmias during ischemia

In this model of regional ischemia, severe ventricular arrhythmias peaked after 7 to 15 minutes of coronary artery occlusion.

### Incidences of VT or VF

**Figure 2:**
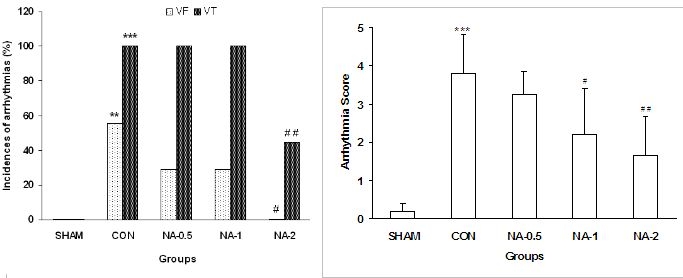
**A:** The incidence of VF and VT **B:** Distribution of the arrhythmia score during 25 min ischemia in SHAM, Control (CON) and different doses of noradrenaline (NA-0.5, NA-1 and NA-2) groups. *** p<0.001 and ** p<0.01 vs. sham group. ## p<0.01 and # p<0.05 vs. control group.

Surgical procedures were not led to occurrence of VT or VF in sham-operated rats and VEBs only displayed in many hearts. In the control non-preconditioned hearts, VT was observed in 100% of rats and 55.5 % of the hearts displayed VF. Pretreatment with 0.5 or 1 μg/kg of noradrenaline had no effect on incidences of VF and VT during 25-min ischemia. On the other hand, administration of high-dose of noradrenaline prior to ischemia, attenuated VT to 44.5% and totally abolished VF compared to the saline control rats (Figure 2A).

### Severity of arrhythmias

Coronary artery occlusion increased the severity of arrhythmias in the saline-treated control compared to sham-operated rats. Compared to control, severity of ventricular arrhythmias significantly declined by injection of noradrenaline (1 or 2 μg/kg) prior to ischemia (2.2±1.5 and 1.7±1.2 vs. 3.9±0.9 in control rats). Injection of 0.5 μg/kg of noradrenaline had no protective effect on severity of ischemia-induced arrhythmias (3.25±0.6) (Figure 2B).

It was revealed that significantly, there was an inverted correlation between pretreatment with different doses of noradrenaline and severity of arrhythmias during 25-min ischemia (R= - 0.75, p<0.01) (Figure 2B).

### Number of episodes of VEB/ min

**Figure 3:**
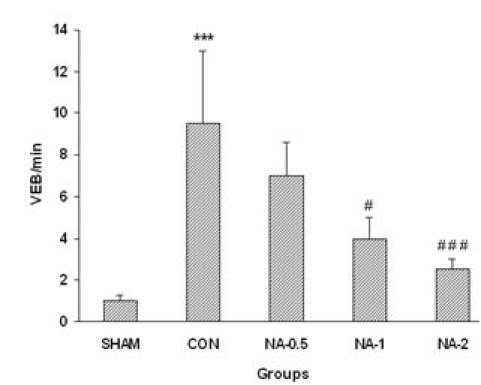
Number of episodes of ventricular ectopic beats/min (VEBs/min).Different doses of noradrenaline (NA-0.5, NA-1and NA-2). Data are presented as mean ± SD. *** p<0.001 vs. Sham. # p<0.05 and ### p<0.001 vs. control group.

Compared to control, number of episodes of VEB/min significantly improved by injection of noradrenaline (1 or 2 μg/kg) prior to ischemia (4±1 and 2.5±1.2 vs. 9.5±4.5 in control rats). Injection of 0.5 μg/kg of noradrenaline had no protective effect on number of episodes of VEB/min (7±1.6) (Figure 3).

### Corrected QT (QTc) interval

The change in QTc interval (ms) was measured between the baseline and 25-min ischemia periods within groups (Table 2). In the saline control rats, the induction of 25-min ischemia significantly reduced the QTc interval compared to the baseline period (p<0.05). By administration of 0.5 or 1 μg/kg of noradrenaline, the QTc interval during prolonged ischemia was not significantly changed compared to the baseline period. Injection of 2 μg/kg of noradrenaline significantly increased the QTc interval (p<0.05) during prolonged ischemia compared to the baseline period.

## Discussion

In this study, noradrenaline dose-dependently prolonged the QTc interval and reduced severity and incidences of ventricular arrhythmias.

The protection does not appear to be due to an increase in coronary artery flow because noradrenaline administration did not enhance MBP in 25-min ischemia period and similar to other groups, occlusion of LAD led to a fall in MBP.

The heart rate is another parameter that might play a part in arrhythmogenesis [17]. In our study, there was no difference in the heart rate during ischemia period among the groups, and then it seems that the heart rate had no effect on arrhythmogenesis.

The QT interval is affected by heart rate and to assess the QT interval independently of heart rate, it is expressed as a corrected QT (QTc). It is generally believed that the QT interval is determined by the action potential duration (APD) of the ventricular myocytes [18]. It is notable that ischemia-induced arrhythmias originate from the boundary zone between the normal region and ischemic area. In the boundary zone, shortening of APD during ischemia will be accompanied by a shortening of refractoriness, which would be proarrhythmic [19].

In our experiment, 25-min regional ischemia shortened the QTc interval and induced ventricular arrhythmias. Administration of different doses of noradrenaline prevented the shortening of QTc interval during ischemia and only injection of maximum dose of noradrenaline (2μg/kg) significantly prolonged the QTc interval and reduced severity and incidence of ventricular arrhythmias. Therefore, it seems that antiarrhythmic effect of noradrenaline is very similar to many related drugs such as amiodarone [20]. This effect most probably mediates via QTc prolongation.

It is proposed that administration of catecholamine prolongs the refractoriness and APD via the α1-adrenoreceptor activation [21]. In an earlier study, we concluded the contribution of the α1-adrenoreceptors to the protection of noradrenaline-induced preconditioning [22]. Therefore, it seems that noradrenaline by prolonging the QTc interval, action potential duration and subsequent refractoriness could improve the ischemia-induced ventricular arrhythmias.

It is assumed that in ischemic myocardium, catecholamines are involved in worsening of arrhythmias by increasing automaticity and triggered activity [23], but it has been shown that there is no relationship between circulating catecholamines and occurrence of arrhythmias in clinical [24] and experimental studies  [25]. In our study, noradrenaline pretreatment could improve ischemia-induced ventricular arrhythmia in the anesthetized rat heart as we expected.

Administration of catecholamine has been shown to inhibit Na+ accumulation and K+ loss during myocardial ischemia [26], which maybe the reason for ischemia-induced ventricular arrhythmias suppression by noradrenaline.

In general, the accessible evidence supports the idea that preconditioning within clinical procedures may be advantageous to patients. However, α1-antagonists that are now recommended as one of the first-line drugs in the management of hypertension [27] could be potentially deleterious agents that may either abolish or prevent the development of preconditioning. It has been shown that the use of prazosin (a selective α1-antagonist) increases the number of chest pain episodes and ST-segment deviations in ECG from the baseline in the patients with unstable angina. On the other hand, it is possible that β-blocker-induced reduction in mortality and morbidity in patients with myocardial infarction [28] may also be mediated by induction of cardioprotection by allowing endogenous noradrenaline to activate α1- adrenoceptors.

## Conclusion

Noradrenaline dose-dependently attenuated ischemia-induced arrhythmias in the rat heart in vivo.

## Figures and Tables

**Table 1:**
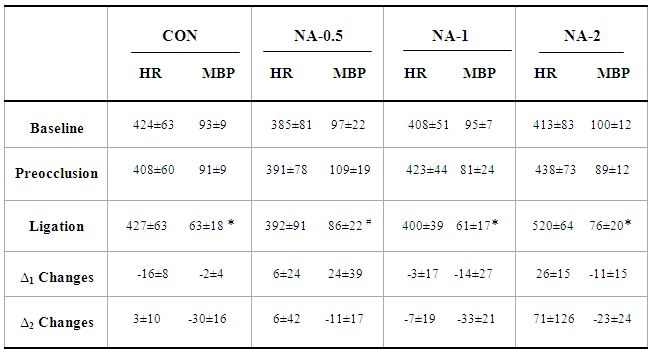
Hemodynamic parameter

HR: Heart Rate (beats/min), MBP: Mean Arterial Blood Pressure (mmHg), NA: Noradrenaline. Data are presented as mean ± SD. * p<0.05 and ** p<0.01 compared with baseline within group and # p<0.05 compared with preocclusion within group. ∆1 changes: differences between preocclusion and baseline periods. ∆2 changes: differences between ligation and baseline periods.

**Table 2:**
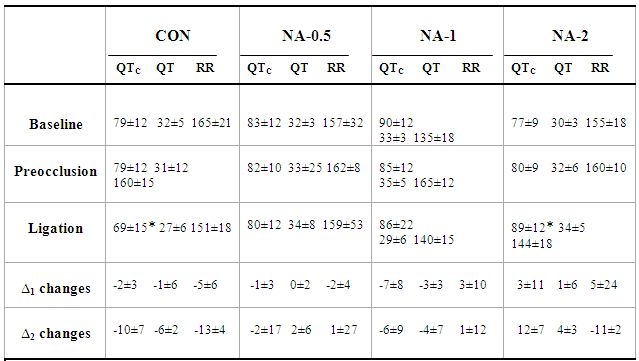
Electrocardiogram Parameters

NA: Noradrenaline, QTc: corrected QT interval (ms). Data are presented as mean ±SD. * p<0.05 compared to baseline within groups. ∆1 changes: differences between preocclusion and baseline periods. ∆2 changes: differences between the ligation and baseline periods.
